# Feasibility of Using Games to Improve Healthy Lifestyle Knowledge in Youth Aged 9-16 Years at Risk for Type 2 Diabetes: Pilot Randomized Controlled Trial

**DOI:** 10.2196/33089

**Published:** 2022-06-17

**Authors:** Ralph Maddison, Nilufar Baghaei, Amanda Calder, Rinki Murphy, Varsha Parag, Ihirangi Heke, Rosie Dobson, Samantha Marsh

**Affiliations:** 1 Institute for Physical Activity and Nutrition Deakin University Geelong Australia; 2 National Institute for Health Innovation University of Auckland Auckland New Zealand; 3 Games and Extended Reality Lab Massey University Auckland New Zealand; 4 School of Information Technology and Electrical Engineering The University of Queensland Brisbane Australia; 5 Department of Medicine Middlemore Hospital University of Auckland Auckland New Zealand; 6 Heke Consulting Auckland New Zealand

**Keywords:** children’s health, diabetes mellitus, type 2 diabetes, experimental games, recruitment

## Abstract

**Background:**

Mobile games can be effective and motivating tools for promoting children’s health.

**Objective:**

We aimed to determine the comparative use of 2 prototype serious games for health and assess their effects on healthy lifestyle knowledge in youth aged 9-16 years at risk for type 2 diabetes (T2D).

**Methods:**

A 3-arm parallel pilot randomized controlled trial was undertaken to determine the feasibility and preliminary effectiveness of 2 serious games. Feasibility aspects included recruitment, participant attitudes toward the games, the amount of time the participants played each game at home, and the effects of the games on healthy lifestyle and T2D knowledge. Participants were allocated to play *Diabetic Jumper* (n=7), *Ari and Friends* (n=8), or a control game (n=8). All participants completed healthy lifestyle and T2D knowledge questionnaires at baseline, immediately after game play, and 4 weeks after game play. Game attitudes and preferences were also assessed. The primary outcome was the use of the game (specifically, the number of minutes played over 4 weeks).

**Results:**

In terms of feasibility, we were unable to recruit our target of 60 participants. In total, 23 participants were recruited. Participants generally viewed the games positively. There were no statistical differences in healthy lifestyle knowledge or diabetes knowledge over time or across games. Only 1 participant accessed the game for an extended period, playing the game for a total of 33 min over 4 weeks.

**Conclusions:**

It was not feasible to recruit the target sample for this trial. The 2 prototype serious games were unsuccessful at sustaining long-term game play outside a clinic environment. Based on positive participant attitudes toward the games, it is possible to use these games or similar games as short-term stimuli to engage young people with healthy lifestyle and diabetes knowledge in a clinic setting; however, future research is required to explore this area.

**Trial Registration:**

Australia New Zealand Clinical Trials Registry ACTRN12619000380190; https://www.anzctr.org.au/Trial/Registration/TrialReview.aspx?id=377123

## Introduction

As the prevalence of type 2 diabetes (T2D) in New Zealand’s younger population increases, the challenge of designing effective and engaging ways and means of preventing the disease also increases [1,2]. Recently, there has been an upsurge of interest in the role of using computer and video games to enhance health outcomes for youth [3,4]. These “serious video games” are designed to entertain players, as they educate, train, or change behavior [3]. For example, games for health are serious video games focused on health [5], with most having some positive outcomes [5], such as effectively promoting dietary change among youth [6]. Indeed, existing video games have helped children between the ages of 10 and 12 years make healthier diet and physical activity choices, with children as young as 9 years understanding the games [7]. Games for health could help young people with T2D attain better self-management by making education about healthy lifestyles more engaging and fun [8]. However, there is a dearth of games designed specifically for youth with T2D [5].

One game that was developed to promote knowledge of the interaction of physical activity behaviors and diet with blood glucose, and knowledge of blood glucose monitoring in young people with T2D resulted in promising outcomes [9,10]. In a preliminary pre-post pilot trial, 12 children aged 9-13 years without T2D or prior experience playing videogames found the game to be fun. Moreover, their engagement was high, and they felt part of a creative and dynamic game community. Importantly, the game was found to enhance the children’s knowledge of healthy diet and lifestyle choices [9,10]. By taking into account user preferences and abilities from this prototype game, we developed 2 new serious games for health. We undertook a pilot randomized controlled study to determine the feasibility and preliminary effectiveness of the 2 serious games. Specifically, the feasibility aspects were recruitment, participant acceptability, preferences and attitudes toward the game, and the amount of time participants played each game in their own environments.

## Methods

### Study Design and Participants

A 3-arm parallel pilot randomized controlled trial (RCT) was undertaken in Auckland, New Zealand between April and September 2019. Eligible participants were aged between 9 and 16 years, had a family history of T2D, were overweight or obese for their age according to the Cole International cutoff points for BMI, or had been told by their doctor that they were at risk for T2D. Eligible participants were also required to have access to an Android device, be able to provide assent (if under the age of 16 years) or consent (if 16 years or older), be able to speak and understand English, and live in the Auckland region. Eligible parents or caregivers were over the age of 18 years, could provide written informed consent on behalf of the child (if the child was under 16 years old), and could speak and understand English.

### Ethics Approval

Ethics approval was obtained from the University of Auckland Human Participants Ethics Committee (ref# 022616). The Consolidated Standards of Reporting Trials (CONSORT) guidelines for reporting parallel group RCTs were followed [11] (Multimedia Appendix 1).

### Recruitment

Participants were recruited from April to September 2019 through posts on websites and social media (Neighbourly, Facebook, and Twitter) by the University of Auckland and affiliates, and paid Facebook and Google advertisements that targeted Auckland parents. Specifically, we included 7 weeks of continuous Facebook advertising, 4 weeks of continuous Google advertising, and 3 to 4 separate posts on Twitter and Facebook. We also placed recruitment flyers in waiting rooms or reception areas of community centers and businesses in the health or youth sector (eg, Waitakere Foot Clinics, YMCA, Sport Auckland, and ProCare-associated general practices). Participants were also recruited through face-to-face contact by the research assistant attending 14 separate community events and talking to parents, or through a trusted third party such as a community program. After 3 months of low recruitment, our approach was updated to include schools as potential third parties. Three schools were approached, and one consented to be involved. Further, the steering committee made 3 amendments to the trial in a bid to reduce barriers to participation and meet recruitment targets. We updated the study eligibility criteria so that participants no longer required access to an Android device and were no longer restricted to living in the Auckland region. Additionally, advertising materials no longer mentioned that we were targeting overweight/obese children for this intervention [12]. These amendments were supplemented by approaching more than 30 potential third parties, including New Zealand teachers (on the Facebook page), 9 Christchurch schools, researchers from other New Zealand Universities, and Diabetes NZ and affiliated clinics.

### Procedure

The parent or caregiver and child attended a 1-hour clinic-style study assessment with a trained research assistant, which was held at the National Institute for Health Innovation, Auckland or at the child’s school. The child completed questions online pertaining to a healthy lifestyle and T2D (Multimedia Appendix 2) before being allocated at random to play 1 of 3 games: *Ari and Friends*, *Diabetic Jumper*, and *Doodle Jump* (control game). Participants were randomized in a 1:1:1 ratio, using a computer-generated random sequence with block randomization involving block sizes of 3 created by the study statistician. Immediately after the initial game play and 4 weeks after the meeting, the children completed the online healthy lifestyle and T2D questionnaires again, with additional questions on game attitudes and preferences (Multimedia Appendix 3). Participants were invited to play the game over the 4 weeks as much as they liked in between assessments. Game play was recorded by the game software. All participants were given two NZ $40 (US $26) supermarket vouchers: one (in person) at baseline and the other (through post) at follow-up.

#### Intervention

**Figure 1:**
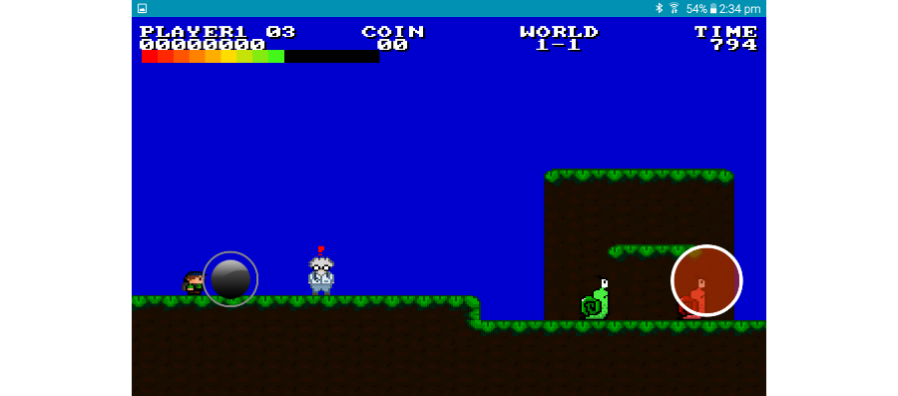
In-game image from *Ari and Friends*.

**Figure 2:**
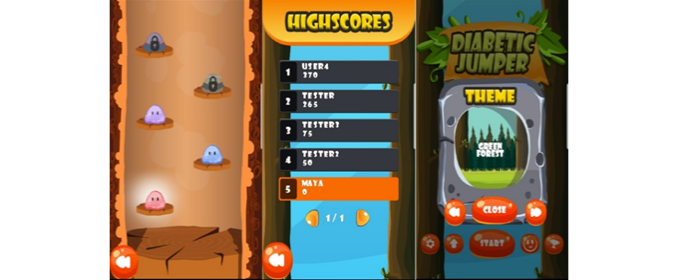
In-game image from *Diabetic Jumper*.

The first game *Ari and Friends* was implemented in Android and was adapted from a Mario Brothers open-source platform (Figure 1), and the second game *Diabetic Jumper* (also in Android) was adapted from a *Doodle Jump* open-source platform (Figure 2). The research team modified concepts from the respective games to suit this project. Initial development involved prototyping the game features with the research team and some tertiary students before formal evaluation was undertaken. Using user-design principles, we recruited a convenience sample of potential end users (children of the same age as the trial) who played early iterations of the respective games and provided feedback to developers and the research team in a workshop format. In total, there were 4 such iterative development workshops prior to the final pilot study, and they were based on feedback that users were happy playing the final versions of the games on the mobile platforms.

**Figure 3:**
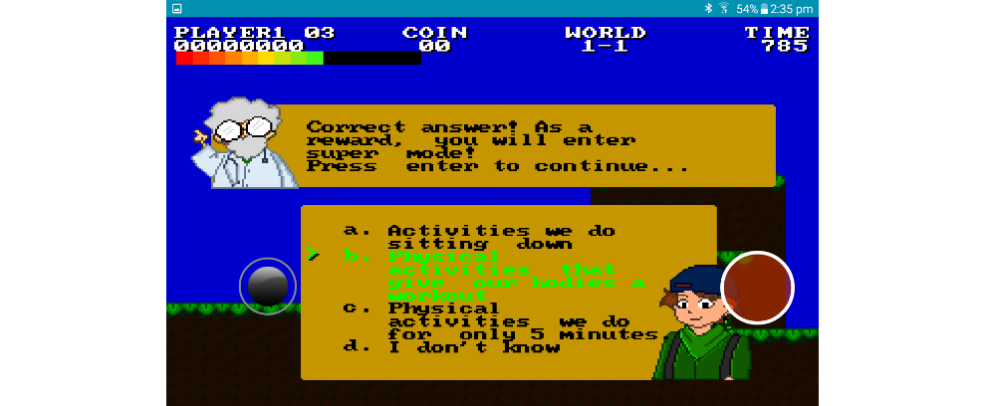
In-game example of the questions asked and answered in *Ari and Friends*.

Both of the final games included features designed to increase children’s knowledge of energy intake and expenditure. For example, by selecting certain foods as the player progressed through the levels, they began to understand the impact of physical activity (stamina to run or jump) and of sweet foods and drinks on their energy levels. Moreover, questions about healthy lifestyle and T2D were interspersed in the game (Multimedia Appendix 4), which could boost power levels if they answered correctly (Figure 3). Players were informed if their answers were correct, and if not, the correct answer was highlighted. For example, at level 1 of the game, children were asked, “How much moderate and/or vigorous physical activity should you do in a week?” Various options were then provided (eg, “at least half an hour a day at least 3 times a week” or “at least 1 hour a day every day of the week”), which the child could select. Children could play the game for a maximum of 15 min per session.

#### Control

The control game *Doodle Jump* did not have any information on T2D or healthy lifestyle behaviors. The object of the game was similar to *Diabetic Jumper*, with the aim to use platforms to continue jumping upwards without falling. Children allocated to the control game also played for 15 min per session.

### Outcomes

The primary outcome was the number of hours played over a 4-week period recorded via the game software. Secondary outcomes were assessed via questionnaires 15 min (±5 min) after game play during the baseline session and at the 4-week follow-up. Secondary outcomes included (1) change in healthy lifestyle knowledge from baseline, (2) change in diabetes-specific knowledge from baseline, and (3) attitudes and preferences assessed using open-ended and free-text questions regarding the games. The questionnaires were designed specifically for this study and contained the same healthy lifestyle and T2D questions that boosted power levels in the games. Specifically, there were 18 multiple-choice questions about healthy lifestyles (specifically about physical activity, screen time, sleep, sedentary behavior, sources of health information, and nutrition) and 9 true/false questions about T2D. Questions about specific healthy lifestyle topics featured in different levels of the 2 intervention games. For example, level 1 in both games contained questions about physical activity and diabetes, level 2 contained questions about nutrition and diabetes, and the remaining 4 levels contained other healthy lifestyle topics. See Multimedia Appendix 4 for the full list of questions.

### Statistical Analysis

Our recruitment target was 60 participants, with 20 participants per study arm. As this was a pilot trial, our recruitment target was not powered to detect significant differences between groups. Study data were collected using REDCap, and all analyses were performed using SAS version 9.4 (SAS Institute). Baseline and follow-up variables were summarized according to group and descriptive summary statistics provided. The change from baseline in continuous outcomes was analyzed using ANOVA for normally distributed data and the Kruskal-Wallis test for nonnormally distributed data.

## Results

### Participants

In terms of feasibility, we were unable to recruit our target of 60 participants. In total, 23 participants (mean age 11 years, SD 1 year) were recruited between April and September 2019 through direct methods (Multimedia Appendix 5). Four participants were recruited via advertisements, while 19 participants were recruited from 1 Auckland school. Seven children were randomized to *Diabetic Jumper*, 8 to *Ari and Friends*, and 8 to the control game. Participants identified predominantly as Samoan (n=15) and Tongan (n=6), and their BMI ranged from 22 to 46 kg/m^2^. See Table 1 for details.

**Table 1:** Participant demographic characteristics.

Characteristic	Intervention: *Diabetic Jumper* (n=7)	Intervention: *Ari and Friends* (n=8)	Active control (n=8)
Age (years), mean (SD)	11 (1.1)	11.3 (1.4)	11.3 (2.9)
Sex (female), n (%)	2 (30)	5 (60)	7 (90)
Weight (kg), mean (SD)	71.8 (23.3)	88.3 (29.0)	84.4 (29.2)
Height (m), mean (SD)	1.52 (0.1)	1.56 (0.1)	1.56 (0.3)
BMI (kg/m^2^), mean (SD)	29.9 (7.2)	35.5 (8.7)	33.6 (6.9)
Ethnicity (Samoan), n (%)	4 (57)	4 (50)	7 (88)

### Game Play

All participants played the games during the initial visit (mean duration 15 min, SD 2 min). Only 1 participant accessed the game after the testing session. The participant played *Diabetic Jumper* thrice in the first evening and twice the next day (total 33 min).

### Diabetes Knowledge

The mean correct scores for diabetes knowledge ranged from 4.6 to 6.6 (51%-73%) for each game at baseline, 5.6 to 6.0 (62%-66%) at the 15-min follow-up, and 5.3 to 6.6 (58%-73%) at the 4-week follow-up (Table 2). There was no statistically significant difference in the mean change in the diabetes knowledge score at the 15-min follow-up (*P*=.93) or the 4-week follow-up (*P*=.10).

**Table 2:** Mean correct scores in the diabetes and healthy lifestyle knowledge questionnaires across the 2 intervention games (*Ari and Friends* and *Diabetic Jumper*) and the control game.

Outcome		Game										*P* value	
		*Ari and Friends*			*Diabetic Jumper*			Control game					
		N	Score^a^, mean (SD)	N		Score^a^, mean (SD)	N		Score^a^, mean (SD)				
**Diabetes knowledge questionnaire (maximum score=9)**													
	Baseline	8	6.6 (1.4)	7		4.6 (1.0)	8		5.8 (2.6)				
	15-min follow-up	8	5.6 (1.4)	7		5.6 (1.5)	8		6.0 (1.8)				
	4-week follow-up	8	5.3 (0.9)	7		5.4 (2.1)	8		6.6 (1.3)				
	Change (15-min − baseline)	8	−1.0 (1.7)	7		1.0 (1.7)	8		0.3 (1.7)	.93			
	Change (4-week − baseline)	8	−1.4 (1.5)	7		0.9 (2.7)	8		0.9 (2.5)	.10			
**Healthy lifestyle knowledge questionnaire (maximum score=18)**													
	Baseline	8	9.4 (2.7)	7		10.0 (3.1)	8		10.5 (3.1)				
	15-min follow-up	8	10.4 (3.1)	7		10.6 (3.3)	8		11.4 (3.2)				
	4-week follow-up	8	9.1 (2.2)	6		11.3 (1.9)	8		10.6 (3.6)				
	Change (15-min − baseline)	8	1.0 (2.0)	7		0.6 (1.4)	8		0.9 (1.1)	.86			
	Change (4-week − baseline)	8	−0.3 (1.4)	6		0.8 (2.1)	8		0.1 (2.4)	.64			

^a^Higher scores indicate a better outcome (more correct answers).

### Healthy Lifestyle Behavior Knowledge

The mean correct scores for healthy lifestyle behavior knowledge ranged from 9.4 to 10.5 (52%-58%) for each game at baseline, 10.4 to 11.4 (57%-63%) at the 15-min follow-up, and 9.1 to 11.3 (50%-63%) at the 4-week follow-up (Table 2). There was no statistically significant difference in the mean change in the healthy lifestyle behavior knowledge score at the 15-min follow-up (*P*=.86) or the 4-week follow-up (*P*=.64).

### Attitudes and Preferences at the 15-min Follow-up

For *Ari and Friends*, 5 of the 8 (63%) participants indicated that the controls were easy to use and that the game was fun, but only 2 of the 8 (25%) participants said that they would recommend the game to their friends.

For *Diabetic Jumper*, 6 of the 7 (85%) participants indicated that the controls were easy to use and the game was fun, and 3 of the 7 (42%) participants indicated that they would recommend the game to their friends.

Overall, 16 of the 23 (69%) participants reported that their parents allowed them to play video games in general “a bit” or “a lot,” and 13 of the 23 (56%) participants reported that their parents restricted their video game play to some extent. Moreover, 10 of the 23 (43%) participants were unsure if their parents would allow them to play the games from the study.

## Discussion

### Principal Findings

Overall, this pilot trial sought to determine the feasibility and preliminary knowledge effects of using 2 prototype serious games for health to improve healthy lifestyle knowledge in youth aged 9-16 years at risk for T2D. The results showed no evidence of improved healthy lifestyle or diabetes knowledge immediately after playing the games or after 4 weeks. Despite all participants playing the games for the allocated 15 min and the mostly positive feedback about each of the 2 games, only 1 participant played the game after leaving the clinic, and this participant played only during the following 2 days. These results coupled with low participant numbers indicated that the games were not engaging enough to result in sustained play and that the intervention methodology was not feasible. However, there may still be potential for using these games in a clinic setting as tools with which to engage youth in healthy lifestyle and T2D knowledge alongside individual clinical advice.

### Comparison With Prior Work

The operation of the 2 prototype games and the method of data extraction from the games were successful. There were no technical issues experienced in either of the games, and extraction of data on play duration, question answers, and food choices from the game software was achieved without any issues. Moreover, the children enjoyed playing both games. In combination, these 3 outcomes indicated that the games were reliable tools to briefly engage with young people who are at risk of diabetes or with diabetes. These outcomes align with the purpose of diabetes-based games for health, making the learning of glycemic control practices engaging and fun [8]. Contrary to previous games for health research, however, there was no evidence from this pilot trial that playing either of the games led to a change in the knowledge of healthy lifestyle or diabetes in the short or long term. This outcome was unexpected as many health-related video games have demonstrated positive outcomes [6]. Indeed, some games for health have resulted in children making positive changes in their diet and physical activity choices [7]. It was difficult to determine whether the lack of effects of these games was a result of the games themselves or a lack of statistical power to detect a change in knowledge over time. Unfortunately, the trial was hampered by recruitment issues. Despite extensive efforts to recruit participants, less than 40% of the recruitment target was achieved over 7 months.

Previous studies involving games for health with hard-to-reach populations, such as youth at risk for diabetes, have ranged from case studies (n=1) to large experimental studies (n=200) [4]. Larger studies have reported using the same recruitment methods as those in this trial, for example, recruiting entire schools [13], collaborating with third parties (eg, recruiting diabetes clinics to gain access to adolescents with diabetes [14,15] and having the trial endorsed by a trusted and known staff member in Māori communities [16]), and using online communication tools and social media platforms [10,17,18]. Of note is that many of these studies did not report the initial recruitment target or the detailed recruitment methods [19,20].

We propose 2 primary reasons for why the recruitment methods in this trial were not as successful as those in previous trials involving games for health. First, there was a teacher strike [21] and a national measles outbreak during the recruitment period. As a result, running a trial may not have been attractive/practical for teachers during this time. Second, it takes time to build a trusting and respectful relationship between communities (eg, schools and health organizations) and research institutes [22]. Over-researched communities are increasingly wary of potential negative effects from having institutes (outsiders) conduct research [23]. Potential negative effects can be negated through including community members in lead roles (which has improved community self-efficacy through problem solving and making decisions) and promoting independence from the researcher by providing tools and resources to the community once the trial is complete [16,24]. Positive relations have also developed between research institutes and communities when health research projects are co-designed [25]. We believe that public health researchers would benefit from investing time and resources into building strong relationships with third parties before designing a trial. Future research should consider a long notice period when planning recruitment, establish relationships with communities and schools, and keep in mind that schools present an annual charter outlining their plans and aims up to 5 years in the future [26].

### Strengths and Limitations

Key strengths of this trial include the considerable formative work to develop the games, the RCT design, and the objective collection of game play directly via the game software. One design limitation that hindered recruitment was having 2 consent processes when recruiting through a school. The school principal consented to conducting research on school grounds during or after school time with the pupils. Due to the age of the participants, parents also consented to participate or for a school or YMCA staff member to be present in lieu of the parent. Consequently, children were responsible for taking the consent forms home to their parents and returning the signed forms to a staff member. Forms were subsequently lost or forgotten and never returned. These consent processes were designed to remove the burden from parents having to attend baseline sessions and to keep their children involved. Unfortunately, the children received the burden of looking after the form. It is possible that recruitment could have been more successful with an alternative process (eg, online forms) to ease the burden on the participant dyad.

Three potential sources of bias should be noted. First, parents, caregivers, or staff members were present during the clinic-type sessions. The presence of authority figures might have influenced participants’ attitudes and preferences toward the games in a bid to be polite, please the authority figure, or behave oneself. This “effort to please” could explain why attitudes and preferences were positive, yet the games were not played during follow-up. Second, the eligibility criteria stipulated that participants had to have access to an Android device, not necessarily that they had to own one themselves. Devices were predominantly owned by parents or elder siblings, and the children might not have the opportunity to use the devices at home, limiting their opportunity to play the games. Third, the research assistant read the questions aloud to many of the younger participants who were struggling to read themselves. The results showed no evidence of change in knowledge, indicating that any unintentional inflection was undetected by the limited sample size or, more likely, was indicative that the participants did not understand the questions at all and guessed.

### Future Directions

The original purpose of the 2 prototype games was for children to play them while waiting with their parents to see the doctor [10]. Initial game play in a waiting room at a diabetes clinic could be a catalyst for discussions among clinicians, parents, and young people about the future risk and management of T2D, including healthy lifestyles. If a future trial is designed to mimic this situation, family members could also play the games. We propose to investigate whether these games can be used to build conversation pathways about T2D and healthy lifestyles between families and clinicians.

### Conclusion

It was not feasible to recruit the target sample for this trial. Our 2 prototype serious games were unsuccessful at sustaining long-term play outside a clinic environment. Based on positive participant attitudes toward the games, it is possible to use these games or similar games as short-term stimuli to engage young people with healthy lifestyle and diabetes knowledge in a clinic setting; however, future research is required to explore this area.

## Appendix

Multimedia Appendix 1 CONSORT checklist.

Multimedia Appendix 2 Type 2 diabetes and lifestyle questions.

Multimedia Appendix 3 Follow-up questionnaire.

Multimedia Appendix 4 Embedded game questions.

Multimedia Appendix 5 CONSORT recruitment diagram.

## Abbreviations

T2D: type 2 diabetes
RCT: randomized controlled trial

## References

[1] Ministry of Health Living Well with Diabetes: A plan for people at high risk of or living with diabetes 2015–2020. Ministry of Health, New Zealand.

[2] Jefferies C, Carter P, Reed PW, Cutfield W, Mouat F, Hofman PL, Gunn AJ (2012). Pediatr Diabetes.

[3] Stokes B (2005). Videogames have changed: time to consider ‘Serious Games’?. The Development Education Journal.

[4] Sardi L, Idri A, Fernández-Alemán JL (2017). A systematic review of gamification in e-Health. J Biomed Inform.

[5] Baranowski T, Buday R, Thompson DI, Baranowski J (2008). Playing for real: video games and stories for health-related behavior change. Am J Prev Med.

[6] Baranowski T, Baranowski J, Cullen KW, Marsh T, Islam N, Zakeri I, Honess-Morreale L, deMoor C (2003). Squire’s Quest!. American Journal of Preventive Medicine.

[7] Baranowski T, Baranowski J, Thompson D, Buday R, Jago R, Griffith MJ, Islam N, Nguyen N, Watson KB (2011). Video game play, child diet, and physical activity behavior change a randomized clinical trial. Am J Prev Med.

[8] Chen G, Baghaei N, Sarrafzadeh A, Manford C, Marshall S, Court G (2011). Designing games to educate diabetic children. OzCHI '11: Proceedings of the 23rd Australian Computer-Human Interaction Conference.

[9] Baghaei N, Nandigam D, Casey J, Direito A, Maddison R (2015). Evaluating mobile games for diabetes education. Proceedings of the 23rd International Conference on Computers in Education, ICCE 2015.

[10] Baghaei N, Nandigam D, Casey J, Direito A, Maddison R (2016). Diabetic Mario: Designing and Evaluating Mobile Games for Diabetes Education. Games Health J.

[11] Schulz K, Altman D, Moher D, CONSORT Group (2010). CONSORT 2010 Statement: updated guidelines for reporting parallel group randomised trials. BMC Med.

[12] Pont SJ, Puhl R, Cook SR, Slusser W, SECTION ON OBESITY, OBESITY SOCIETY (2017). Stigma Experienced by Children and Adolescents With Obesity. Pediatrics.

[13] Jones B, Madden G, Wengreen H (2014). The FIT Game: preliminary evaluation of a gamification approach to increasing fruit and vegetable consumption in school. Prev Med.

[14] Cafazzo JA, Casselman M, Hamming N, Katzman DK, Palmert MR (2012). Design of an mHealth app for the self-management of adolescent type 1 diabetes: a pilot study. J Med Internet Res.

[15] Chu JTW, Wan A, Stewart SM, Ng KT, Lam TH, Chan SS (2018). Recruitment and Lessons Learned from a Community-Based Intervention Program: The Learning Families Project in Hong Kong. Front Public Health.

[16] Hamerton H, Mercer C, Riini D, McPherson B, Morrison L (2014). Evaluating Maori community initiatives to promote healthy eating, healthy action. Health Promot Int.

[17] Marks A, Wilkes L, Blythe S, Griffiths R (2017). A novice researcher's reflection on recruiting participants for qualitative research. Nurse Res.

[18] Ford KL, Albritton T, Dunn TA, Crawford K, Neuwirth J, Bull S (2019). Youth Study Recruitment Using Paid Advertising on Instagram, Snapchat, and Facebook: Cross-Sectional Survey Study. JMIR Public Health Surveill.

[19] Brauner P, Calero Valdez A, Schroeder U, Ziefle M, Holzinger A, Ziefle M, Hitz M, Debevc M (2013). Increase Physical Fitness and Create Health Awareness through Exergames and Gamification. Human Factors in Computing and Informatics. SouthCHI 2013. Lecture Notes in Computer Science, vol 7946.

[20] Lentelink S, Spil A, Broens T, Hermens H, Jones V (2013). Healthy weight game!: Lose weight together.

[21] Webb R (2019). New Zealand teachers on strike. Jacobin.

[22] Baker E (1999). Principles of practice for academic/ practice/community research partnerships. American Journal of Preventive Medicine.

[23] Kerstetter K (2012). Insider, Outsider, or Somewhere Between: The Impact of Researchers’ Identities on the Community-Based Research Process. Journal of Rural Social Sciences.

[24] Simmons D, Voyle JA (2003). Reaching hard-to-reach, high-risk populations: piloting a health promotion and diabetes disease prevention programme on an urban marae in New Zealand. Health Promot Int.

[25] Ni Mhurchu C, Te Morenga L, Tupai-Firestone R, Grey J, Jiang Y, Jull A, Whittaker R, Dobson R, Dalhousie S, Funaki T, Hughes E, Henry A, Lyndon-Tonga L, Pekepo C, Penetito-Hemara D, Tunks M, Verbiest M, Humphrey G, Schumacher J, Goodwin D (2019). A co-designed mHealth programme to support healthy lifestyles in Māori and Pasifika peoples in New Zealand (OL@-OR@): a cluster-randomised controlled trial. The Lancet Digital Health.

[26] School charter overview. New Zealand Ministry of Education.

