# Supplementary material for: Feasibility of Using Games to Improve Healthy Lifestyle Knowledge in Youth Aged 9-16 Years at Risk for Type 2 Diabetes: Pilot Randomized Controlled Trial
Source: JMIR Form Res. 2022 Jun 17;6(6):e33089. doi: 10.2196/33089 (PMC9250061; doi:10.2196/33089)
Supplement: Multimedia Appendix 2 [file formative_v6i6e33089_app2.pdf]

Form C - Before Playing the Game

Hi there,

Thank you for signing up to Games for Health.

Please answer all of the questions to begin the study.

Thank you!

1 - Diabetes Knowledge

Please select whether each of the following statements are True or False

1.01 - All of the following can increase your risk of getting type 2 diabetes

* Being overweight or obese
* Not doing enough physical activity
* Other people in your family having type 2 diabetes

○ True
○ False

1.02 - People with type 2 diabetes create too much insulin

○ True
○ False

1.03 - Insulin helps to keep your body's blood sugar within a normal range

○ True
○ False

1.04 - For many people, the best way to prevent type 2 diabetes is through a healthy lifestyle

○ True
○ False

1.05 - Type 2 diabetes only occurs in adults

○ True
○ False

1.06 - Everyone with type 2 diabetes has symptoms

○ True
○ False

1.07 - Feeling thirsty may be a symptom of type 2 diabetes

○ True
○ False

1.08 - Feeling like you have lots of energy may be a symptom of type 2 diabetes

○ True
○ False

1.09 - Sometimes when you have type 2 diabetes, cuts and grazes may take longer to get better

○ True
○ False

2 - Healthy Lifestyle Behaviours Knowledge

2.01 - How much moderate-to-vigorous physical activity should you do per week?

○ At least half an hour a day at least 3 times per week
○ At least 1 hour a day every day of the week
○ At least 1 hour per day at least 3 times per week
○ At least half an hour a day every day of the week

2.02 - Which feeling lets you know you are doing exercise that is good for your health?

○ Getting dizzy
○ Becoming sleepy
○ Breathing harder
○ I don't know

2.03 - When you play or exercise, do you think it is better for you if you breathe hard and your heart beats faster?	◯ Yes<br>◯ No
2.04 - What are moderate to vigorous physical activities (tick one only)?	◯ Activities we do sitting down<br>◯ Physical activities that give our bodies a workout<br>◯ Physical activities we do for only 5 minutes<br>◯ I don't know
2.05 - Which ones are moderate to vigorous physical activities (tick all that apply)?	☐ Tag or bulrush<br>☐ Sitting down doing homework<br>☐ Jogging or running<br>☐ Chopping wood<br>☐ Riding a bike<br>☐ I don't know
2.06 - What is the recommended screen time for someone aged 9-15 years?	◯ Less than 1 hour/day<br>◯ Less than 2 hours/day<br>◯ Less than 4 hours/day<br>◯ Unlimited if the screen time is educational
2.07 - If you are getting lots of exercise during the day, is it still important to break up the amount of time you spend sitting?	◯ Yes<br>◯ No
2.08 - Which drinks are recommended for kids (tick all that apply)?	☐ Sugar-free fizzy drinks<br>☐ Water<br>☐ Milk<br>☐ Iced tea<br>☐ Juice
2.09 - Which drink does not have any sugar in it?	◯ Fanta<br>◯ Ice tea<br>◯ Water<br>◯ Orange juice<br>◯ I don't know
2.10 - How do sugar-sweetened drinks affect your health (tick all that apply)?	☐ Increase weight<br>☐ Cause tooth decay<br>☐ Reduce your concentration and attention<br>☐ All of the above
2.11 - How many servings of fruit and vegetables per day are recommended	◯ 1-2<br>◯ 3-4<br>◯ 5 or more
2.12 - What are vegetables high in?	◯ Protein<br>◯ Fats<br>◯ Fibre
2.13 - Who can kids talk to about a health question?	◯ Parent<br>◯ Teacher<br>◯ School nurse<br>◯ Doctor<br>◯ All of the above

2.14 - If you want health information, where is the best place to get it?	◯ Social media<br>◯ Ask a friend<br>◯ Google<br>◯ A nurse or doctor<br>◯ All of the above
2.15 - What helps you sleep at night?	◯ A healthy diet<br>◯ Physical activity during the day<br>◯ Limiting your screen use<br>◯ Having a regular bedtime<br>◯ All of the above
2.16 - How many hours of sleep per night is recommended for children aged 9-13 years?	◯ 7-8 hours<br>◯ 9-11 hours<br>◯ 12 hours<br>◯ I don't know
2.17 - How many hours of sleep per night is recommended for children aged 14-17 years?	◯ 7 hours<br>◯ 8-10 hours<br>◯ 11-12 hours<br>◯ I don't know
2.18 - Does watching TV before bed help you sleep?	◯ Yes<br>◯ No
