# Supplementary material for: Feasibility of Using Games to Improve Healthy Lifestyle Knowledge in Youth Aged 9-16 Years at Risk for Type 2 Diabetes: Pilot Randomized Controlled Trial
Source: JMIR Form Res. 2022 Jun 17;6(6):e33089. doi: 10.2196/33089 (PMC9250061; doi:10.2196/33089)
Supplement: Multimedia Appendix 3 [file formative_v6i6e33089_app3.pdf]

Form E - 1 month Follow-Up

Hi there,

Thank you for taking part in Games for Health and testing our game for us.

Please answer all of the questions below to complete the study.

Thank you!

1 - Diabetes Knowledge

1.01a - Assessment outcome

◯ No Contact
◯ Withdrew
◯ Other

1.01b - If other, please specify

____

1.01c - Please enter today's date:

____

Please select whether each of the following statements are True or False

1.01 - All of the following can increase your risk of getting type 2 diabetes

* Being overweight or obese
* Not doing enough physical activity
* Other people in your family having type 2 diabetes

◯ True
◯ False

1.02 - People with type 2 diabetes create too much insulin

◯ True
◯ False

1.03 - Insulin helps to keep your body's blood sugar within a normal range

◯ True
◯ False

1.04 - For many people, the best way to prevent type 2 diabetes is through a healthy lifestyle

◯ True
◯ False

1.05 - Type 2 diabetes only occurs in adults

◯ True
◯ False

1.06 - Everyone with type 2 diabetes has symptoms

◯ True
◯ False

1.07 - Feeling thirsty may be a symptom of type 2 diabetes

◯ True
◯ False

1.08 - Feeling like you have lots of energy may be a symptom of type 2 diabetes

◯ True
◯ False

1.09 - Sometimes when you have type 2 diabetes, cuts and grazes may take longer to get better

◯ True
◯ False

2 - Healthy Lifestyle Behaviours Knowledge

2.01 - How much moderate-to-vigorous physical activity should you do per week?

- ◯ At least half an hour a day at least 3 times per week
- ◯ At least 1 hour a day every day of the week
- ◯ At least 1 hour per day at least 3 times per week
- ◯ At least half an hour a day every day of the week

2.02 - Which feeling lets you know you are doing an exercise or PA that is good for your health

- ◯ Getting dizzy
- ◯ Becoming sleepy
- ◯ Breathing harder
- ◯ I don't know

2.03 - When you play or exercise, do you think it is better for you (your health) if you breathe hard and your heart beats faster?

- ◯ Yes
- ◯ No

2.04 - What are moderate to vigorous physical activities (tick one only)?

- ◯ Activities we do sitting down
- ◯ Physical activities that give our bodies a workout
- ◯ Physical activities we do for only 5 minutes
- ◯ I don't know

2.05 - Which ones are moderate to vigorous physical activities (tick all that apply)?

- ☐ Tag or bulrush
- ☐ Sitting down doing homework
- ☐ Jogging or running
- ☐ Chopping wood
- ☐ Riding a bike
- ☐ I don't know

2.06 - What is the recommended screen time for someone aged 9-15 years?

- ◯ Less than 1 hour/day
- ◯ Less than 2 hours/day
- ◯ Less than 4 hours/day
- ◯ Unlimited if the screen time is educational

2.07 - If you are getting lots of exercise during the day, is it still important to break up the amount of time you spend sitting?

- ◯ Yes
- ◯ No

2.08 - Which drinks are recommended for kids (tick all that apply)?

- ☐ Sugar-free fizzy drinks
- ☐ Water
- ☐ Milk
- ☐ Iced tea
- ☐ Juice

2.09 - Which drink does not have any sugar in it?

- ◯ Fanta
- ◯ Ice tea
- ◯ Water
- ◯ Orange juice
- ◯ I don't know

2.10 - How do sugar-sweetened drinks affect your health (tick all that apply)?

- ☐ Increase weight
- ☐ Cause tooth decay
- ☐ Reduce your concentration and attention
- ☐ All of the above

2.11 - How many servings of fruit and vegetables per day are recommended

- ◯ 1-2
- ◯ 3-4
- ◯ 5 or more

2.12 - What are vegetables high in?	◯ Protein<br>◯ Fats<br>◯ Fibre
2.13 - Who can kids talk to about a health question?	◯ Parent<br>◯ Teacher<br>◯ School nurse<br>◯ Doctor<br>◯ All of the above
2.14 - If you want health information, where is the best place to get it?	◯ Social media<br>◯ Ask a friend<br>◯ Google<br>◯ A nurse or doctor<br>◯ All of the above
2.15 - What helps you sleep at night?	◯ A healthy diet<br>◯ Physical activity during the day<br>◯ Limiting your screen use<br>◯ Having a regular bedtime<br>◯ All of the above
2.16 - How many hours of sleep per night is recommended for children aged 9-13 years?	◯ 7-8 hours<br>◯ 9-11 hours<br>◯ 12 hours<br>◯ I don't know
2.17 - How many hours of sleep per night is recommended for children aged 14-17 years?	◯ 7 hours<br>◯ 8-10 hours<br>◯ 11-12 hours<br>◯ I don't know
2.18 - Does relaxing watching TV before bed help you sleep?	◯ Yes<br>◯ No

3 - Game feedback

3.01 - Were the game controls easy to use?

◯ No, not at all
◯ No, not really
◯ Not sure
◯ Yes, a bit
◯ Yes, a lot

3.02 - Did you have fun playing the game?

◯ No, not at all
◯ No, not really
◯ Not sure
◯ Yes, a bit
◯ Yes, a lot

3.03 - Do you often play video games?

◯ No, not at all
◯ No, not really
◯ Not sure
◯ Yes, a bit
◯ Yes, a lot

3.04 - Would you play this game in the future?

◯ No, not at all
◯ No, not really
◯ Not sure
◯ Yes, a bit
◯ Yes, a lot

3.05 - Would you recommend the game to your friends?

◯ No, not at all
◯ No, not really
◯ Not sure
◯ Yes, a bit
◯ Yes, a lot

3.06 - Do your parents allow you to play video games?

◯ No, not at all
◯ No, not really
◯ Not sure
◯ Yes, a bit
◯ Yes, a lot

3.07 - Do your parents restrict you playing video games?

◯ No, not at all
◯ No, not really
◯ Not sure
◯ Yes, a bit
◯ Yes, a lot

3.08 - Do you think you play video games too much?

◯ No, not at all
◯ No, not really
◯ Not sure
◯ Yes, a bit
◯ Yes, a lot

3.09 - Would your parent encourage you to play this game?

◯ No, not at all
◯ No, not really
◯ Not sure
◯ Yes, a bit
◯ Yes, a lot

3.10 - How do you think we can improve the game?

____

3.11 - Did you have any problems playing the game?

____

3.12 - Any other comments you wish to make?

____
