# Supplementary material for: Feasibility of Using Games to Improve Healthy Lifestyle Knowledge in Youth Aged 9-16 Years at Risk for Type 2 Diabetes: Pilot Randomized Controlled Trial
Source: JMIR Form Res. 2022 Jun 17;6(6):e33089. doi: 10.2196/33089 (PMC9250061; doi:10.2196/33089)
Supplement: Multimedia Appendix 4 [file formative_v6i6e33089_app4.pdf]

Diabetic Mario Feasibility Trial					
**Game Level**	**Topic**	**Question**	**Answer Options**	**Correct or Incorrect**	**Explanation**
Level 1	Physical Activity	How much moderate and/or vigorous physical activity should you do in a week?	At least 30 minutes a day at least 3 times a week	Incorrect	Aim to engage in at least 60 minutes of moderate to vigorous intensity physical activity on most days of the week
			At least 60 minutes a day on most days of the week	Correct	
			At least 1 hour a day at least 3 times a week	Incorrect	
			At least half an hour a day every day of the week	Incorrect	
		Which feeling lets you know you are doing exercise that is good for your health?	Getting dizzy	Incorrect	Breathing harder as you exercise is good for your health.
			Becoming sleepy	Incorrect	
			Breathing harder	Correct	
			I don't know	Incorrect	
		When you play or exercise, do you think it is better for you if you breathe hard and your heart beats faster?	Yes	Correct	Exercise that makes you breathe harder and your heart beat faster is good for your health.
			No	Incorrect	
		Which one is not a moderate or vigorous physical activity?	Tag or bull rush	Incorrect	Moderate and vigorous physical activities are activities that that make you breathe harder and make your heart beat faster.
			Sitting down doing homework	Correct	
			Jogging or running	Incorrect	
			Chopping wood	Incorrect	
			Riding a bike	Incorrect	
			I don't know	Incorrect	
		True or False? For many people, the best way to prevent type 2 diabetes is through a healthy lifestyle	True	Correct	Getting enough exercise, having a healthy diet, and losing weight are important factors for both preventing and treating type 2 diabetes.
			False	Incorrect	
	Sedentary Behaviour/Screen Time	What is the recommended screen time for someone aged 9-15 years?	Less than 1 hour a day	Incorrect	The recommended amount of screen time for someone aged 9-15 years is less than two hours a day.
			Less than 2 hours a day	Correct	
			Less than 4 hours a day	Incorrect	
			Unlimited if the screen time is educational	Incorrect	
			Yes	Correct	

		If you are getting lots of exercise during the day, is it still important to break up the amount of time you spend sitting?	No	Incorrect	It is still important to break up the amount of time you spend sitting, even if you do a lot of exercise.
		True or False? Feeling like you have lots of energy may be a symptom of type 2 diabetes	True<br>False	Incorrect<br>Correct	Feeling very tired or sleepy may be a symptom of type 2 diabetes.
		Which of the following can increase your risk of getting type 2 diabetes?	Being overweight or obese<br>Not doing enough physical activity<br>Other people in your family having type 2 diabetes<br>All of the above	Incorrect<br>Incorrect<br>Incorrect<br>Correct	Risk factors for diabetes include being obese, not being active enough, and having a family history of type 2 diabetes.
		True or False? Everyone with type 2 diabetes has symptoms	True<br>False	Incorrect<br>Correct	Some people can have type 2 diabetes without any symptoms
Level 3	Sugar	Which drinks are recommended for children?	Sugar-free fizzy drinks<br>Only Water<br>Only Milk<br>Juice<br>Water AND Milk	Incorrect<br>Incorrect<br>Incorrect<br>Incorrect<br>Correct	Water and low-fat Milk are the recommended drinks for children
		Which drink does not have any sugar?	Fanta<br>Iced tea<br>Water<br>Orange juice<br>I don't know	Incorrect<br>Incorrect<br>Correct<br>Incorrect<br>Incorrect	Fizzy drinks, energy drinks, and juice are high in sugar, choose water instead
		How do sugar-sweetened drinks affect your health?	Increase weight<br>Cause tooth decay<br>Reduce your concentration and attention<br>All of the above	Incorrect<br>Incorrect<br>Incorrect<br>Correct	Sugar-sweetened drinks can lead to increased weight, tooth decay, and lower concentration levels.
		Do people with type 2 diabetes create too much insulin or not enough insulin?	Too much<br>Not enough	Incorrect<br>Correct	In T2D, your body does not make enough insulin or doesn't use insulin well.
			True	Correct	

		True or False? Insulin helps to keep your body's blood sugar within a normal range.	False	Incorrect	If the body doesn't produce enough insulin then your blood sugar levels get too high
Level 4	Fruit and Vegetables	How many servings of fruit and vegetables are recommended each day?	1 to 2<br>3 to 4<br>5 or more	Incorrect<br>Incorrect<br>Correct	Eat at least 3 servings of vegetables, and 2 servings of fruit every day
		How many categories of colours do fruit and vegetables fall into?	3<br>5<br>7	Incorrect<br>Correct<br>Incorrect	Fruit and vegetables fall into 5 different categories of colours (red, purple/blue, orange/yellow, green, brown/white)
		What are vegetables high in?	Protein<br>Fats<br>Fibre	Incorrect<br>Incorrect<br>Correct	Vegetables are high in fibre, which is important for our health.
		True or False? Feeling thirsty may be a symptom of type 2 diabetes	True<br>False	Correct<br>Incorrect	Feeling thirsty, going to the toilet often, and often feeling hungry are all symptoms of type 2 diabetes.
		True or False? Type 2 diabetes only occurs in adults	True<br>False	Incorrect<br>Correct	While most cases of diabetes occur in adults, children and adolescents can also get type 2 diabetes.
Level 5	Sleep	What helps you sleep at night?	A healthy diet<br>Physical activity during the day<br>Limiting your screen use<br>Having a regular bedtime<br>All of the above	Incorrect<br>Incorrect<br>Incorrect<br>Incorrect<br>Correct	A healthy diet, physical activity, limiting your screen use and having a regular bedtime all help with sleep.
		How many hours of sleep a night are recommended for children aged 9-13 years?	7-8 hours<br>9-11 hours<br>12 hours<br>I don't know	Incorrect<br>Correct<br>Incorrect<br>Incorrect	Children aged 9-13 years should have 9-11 hours of sleep each night.
		How many hours of sleep a night are recommended for children aged 14-17 years?	7 hours<br>8-10 hours<br>11-12 hours<br>I don't know	Incorrect<br>Correct<br>Incorrect<br>Incorrect	Children aged 14-17 years should have 8-10 hours of sleep each night
			Yes	Incorrect	

		Does watching TV before bed help you sleep?	No	Correct	Screen time before bed does not help you sleep, even if it is relaxing.
Level 6	Health Questions	Who can children talk to if they have a health question?	Parent<br>Teacher<br>School nurse<br>Doctor<br>All of the Above	Incorrect<br>Incorrect<br>Incorrect<br>Incorrect<br>Correct	If you have a health question you can ask your parents, teacher, school nurse or your doctor
		If you want health information, where is the best place to get it?	Social media<br>Ask a friend<br>Google<br>A nurse or doctor<br>All of the above	Incorrect<br>Incorrect<br>Incorrect<br>Correct<br>Incorrect	If you have questions about your health the best person to talk to is a health professional such as a doctor or nurse
		True or False? Sometimes when you have type 2 diabetes, cuts and grazes may take longer to get better.	True<br>False	Correct<br>Incorrect	Both sores and infections that take longer to heal may be a symptom of type 2 diabetes
