# Supplementary figures and images for: Feasibility of Using Games to Improve Healthy Lifestyle Knowledge in Youth Aged 9-16 Years at Risk for Type 2 Diabetes: Pilot Randomized Controlled Trial
Source: JMIR Form Res. 2022 Jun 17;6(6):e33089. doi: 10.2196/33089 (PMC9250061; doi:10.2196/33089)

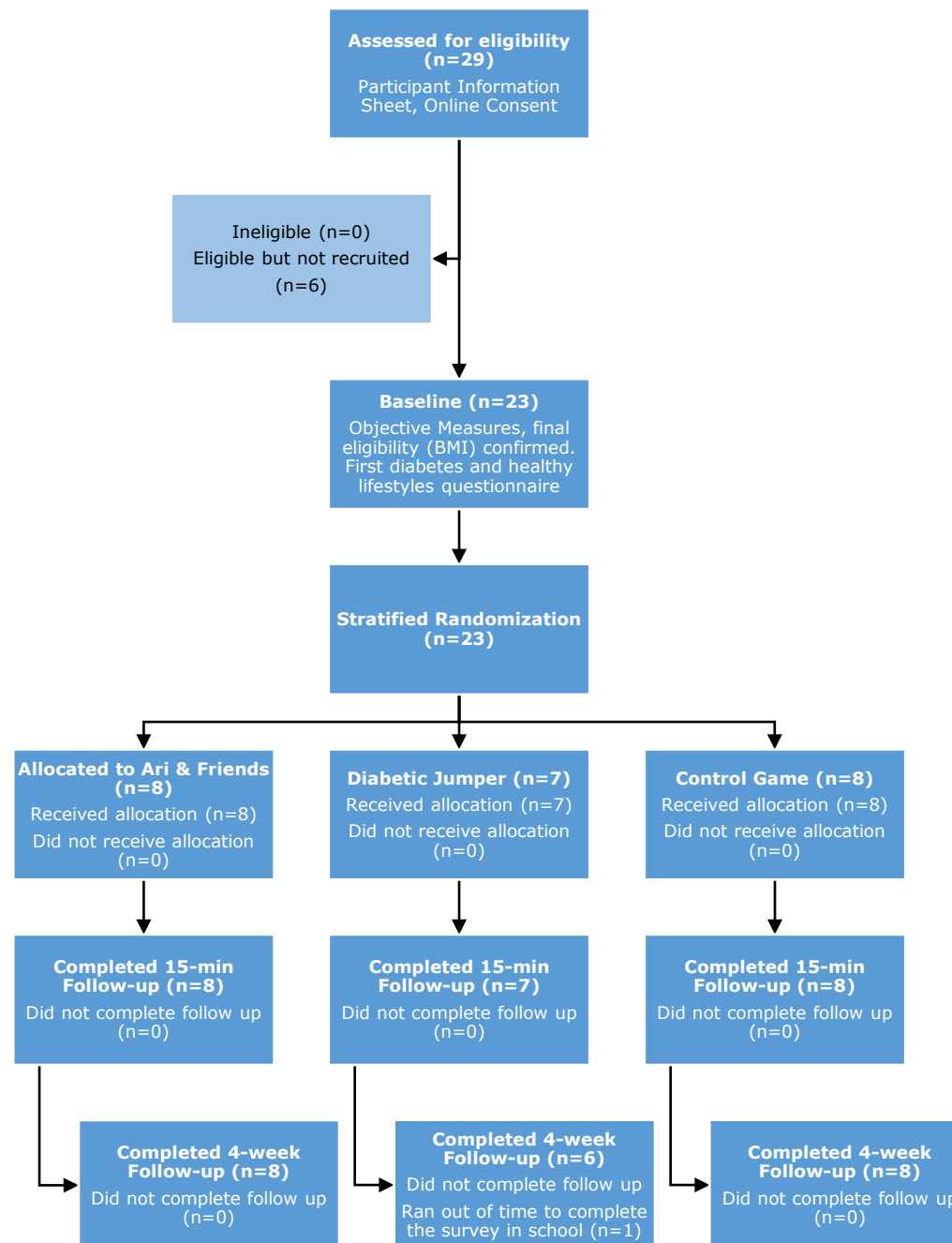

Supplement: Multimedia Appendix 5 [file formative_v6i6e33089_app5.pdf]
